# Comparison of McMaster and Mini-FLOTAC techniques for the diagnosis of internal parasites in pigs

**DOI:** 10.1590/S1984-29612023013

**Published:** 2023-03-27

**Authors:** Camila Souza Carvalho Class, Priscila Alves Fialho, Leucio Câmara Alves, Renato Luiz Silveira, Maria Regina Reis Amendoeira, Fabiana Batalha Knackfuss, Alynne da Silva Barbosa

**Affiliations:** 1 Laboratório de Bioagentes Ambientais, Departamento de Microbiologia e Parasitologia, Instituto Biomédico, Universidade Federal Fluminense – UFF, Niterói, RJ, Brasil; 2 Laboratório de Doenças Parasitárias, Departamento de Medicina Veterinária, Universidade Federal Rural de Pernambuco – UFRPE, Recife, PE, Brasil; 3 Laboratório de Morfologia Animal, Departamento de Morfologia, Instituto Biomédico, Universidade Federal Fluminense – UFF, Niterói, RJ, Brasil; 4 Laboratório de Toxoplasmose e Outras Protozooses, Instituto Oswaldo Cruz, Fundação Oswaldo Cruz – Fiocruz, Rio de Janeiro, RJ, Brasil; 5 Laboratório de Estatística, Universidade do Grande Rio, Duque de Caxias, RJ, Brasil

**Keywords:** Parasitological diagnosis, quantitative techniques, EPG, Nematodes, Diagnóstico parasitológico, técnicas quantitativas, OPG, Nematoide

## Abstract

This purpose of this study was to compare the efficiency of the McMaster and Mini-FLOTAC quantitative techniques in the investigation of helminths in feces of pigs. An analysis was made of 74 fecal samples from pigs raised on family farms located in Rio de Janeiro, Brazil. These were analyzed by the Mini-FLOTAC and McMaster techniques in a solution of 1,200g/mL NaCl. This investigation revealed a superiority in the frequency of all helminths detected by Mini-FLOTAC, including *Ascaris suum*, *Trichuris suis*, strongyles and *Strongyloides ransomi*. The Kappa index revealed substantial agreement in all comparisons made in relation to the frequency of positive samples. However, significant statistical differences in the comparison of EPGs between McMaster and Mini-FLOTAC were observed for all nematodes (p ≤0.05). Higher values of Pearson’s linear correlation coefficient (r), between the techniques in relation to EPG were observed for *A. suum* and *T. suis*, differently from what was observed for strongyles and *S. ransomi*. Mini-FLOTAC proved to be a more satisfactory and reliable technique both for the diagnosis of parasites and for the determination of EPG in pig feces due to the larger size of its counting chambers, thus increasing the helminth egg recovery rates.

## Resumo

Este estudo objetivou comparar a eficiência das técnicas quantitativas de McMaster e Mini-FLOTAC para a pesquisa de helmintos em fezes de suínos. Foram analisadas 74 amostras fecais de suínos mantidos em propriedades familiares, localizadas no Rio de Janeiro, Brasil. Estas foram analisadas pelas técnicas de Mini-FLOTAC e McMaster com solução de NaCl 1.200g/mL. Pode-se verificar uma superioridade na frequência de todos os helmintos detectados por meio do Mini-FLOTAC, incluindo-se *Ascaris suum*, *Trichuris suis,* estrongilídeos e *Strongyloides ransomi*. O índice Kappa mostrou uma concordância substancial em todas as comparações realizadas, em relação à frequência de amostras positivas. Entretanto, diferenças estatísticas significativas na comparação do OPG entre McMaster e Mini-FLOTAC foram observadas para todos os nematoides (p ≤0.05). Elevados valores do coeficiente de correlação de Pearson foram observados entre as técnicas, em relação ao OPG para *A. suum* e *T. suis*, diferentemente do evidenciado para estrôngilos e *S. ransomi.* Mini-FLOTAC mostrou-se uma técnica mais satisfatória e confiável, tanto para o diagnóstico de parasitos quanto para a determinação do OPG em fezes de suínos, devido ao maior tamanho das suas câmaras de contagem, situação que acaba aumentando a eficiência da recuperação dos ovos dos helmintos.

## Introduction

The use of laboratory techniques for helminth egg counting to assess the intestinal parasite load and determine the effectiveness of anthelmintics is common in the field of parasitology, especially in veterinary parasitology. McMaster’s classic quantitative technique for the parasitological examination of feces is one of the most widely used in veterinary medicine for this purpose (Noel et al., 2017; Alowanou et al., 2021). However, new quantitative techniques that are more sensitive have been developed, such as FLOTAC and Mini-FLOTAC (Cringoli et al., 2010, 2017).

The FLOTAC technique is a quantitative centrifugal flotation method that uses two large reading chambers each with a capacity of 5 mL of fecal suspension. This technique is highly sensitive, but it requires the use of a specific centrifuge (Cringoli et al., 2010). On the other hand, the Mini-FLOTAC technique is a variation of the FLOTAC that does not require the use of a centrifuge (Cringoli et al., 2017). The Mini-FLOTAC technique, which involves the passive fluctuation of parasitic structures, allows for the simultaneous diagnosis of helminth eggs/larvae and protozoan oocysts/cysts (Cringoli et al., 2017). In general, studies have demonstrated that the Mini-FLOTAC technique offers higher sensitivity and efficiency than the McMaster technique for research of parasites in samples of humans and other animals (Noel et al., 2017; Dias de Castro et al., 2017; Barda et al., 2014; Alowanou et al., 2021).

However, few studies to date have evaluated the efficiency of this technique in pig feces, that is, the ability to detect the highest number of positive samples and the highest EPG values of helminths. It is known that gastrointestinal parasites have deleterious effects on pigs, causing economic losses for producers, particularly in the case of small farmers for whom these animals represent a source of income and family subsistence (Class et al., 2020). This underscores the importance of estimating the load of parasitic helminths in pigs, since their treatment with anti-parasitic drugs by small farmers is usually haphazard. Therefore, this study involved a quantitative and qualitative comparisons of the efficiency of the McMaster and Mini-FLOTAC parasitological techniques for the detection of helminths in fecal samples from pigs raised on family farms.

## Material and Methods

This study analyzed a total of 74 pig fecal samples equal to or heavier than 20 grams. These animals were of both sexes and different ages and were raised on different family farms located in the municipality of Cachoeiras de Macacu, in the state of Rio de Janeiro, Brazil. The pig feces were collected directly from the rectal ampulla using a long palpation glove lubricated with glycerin. The fecal material thus collected was then sent to the Parasitology Laboratories of the Biomedical Institute at UFF – Federal Fluminense University in isothermal boxes, and immediately processed.

Each fecal sample was processed by the two techniques, Mini-FLOTAC according to Cringoli et al. (2017) and McMaster adapted from Gordon & Whitlock (1939). A Fill-FLOTAC fecal collector designed to extract fecal samples from farm animals was used in both quantitative techniques. The Fill-Flotac® consists of a system consisting of a graduated container with a screw cap that contains a collecting device and a filter already attached.

A standard amount of 5 grams of fecal matter suspended in sodium chloride solution at 1,200 g/mL can be analyzed using this device.

The solution of 45 mL NaCl was added to the Fill-FLOTAC container. Subsequently, the fecal sample was placed in the device collector until it was completely filled, totaling 5 grams of feces. Then, the material was homogenized together with the solution through rotating and vertical movements of the collector. A pipette tip was attached to the device, allowing filling of the Mini-FLOTAC and McMaster chambers directly from the Fill-FLOTAC container.

The two chambers of the devices Mini-FLOTAC and McMaster were read on the same Olympus® BX41 microscope, and as recommended, each sample was read by the same microscopist. After completing each helminth egg count, the result was multiplied by a correction factor of 5 for Mini-FLOTAC and of 33 for McMaster. The parasite load of each detected helminth taxon was classified as recommended by Nwafor et al. (2019), i.e., low when the eggs per gram (EPG) was less than or equal to 100, moderate when the EPG was greater than 100 and less than 500, and high when the EPG was greater than or equal to 500.

The results of the comparison between the Mini-FLOTAC and McMaster quantitative techniques were tabulated and were presented separately, and jointly whenever a helminth was detected by both techniques.

The agreement between the Mini-FLOTAC and McMaster techniques regarding the diagnostic frequency of each helminth taxon detected was determined by Kappa coefficient (k), associated with 95% confidence intervals for its value. The Kappa coefficient as interpreted following the classification of Landis & Koch (1977). Pearson correlation coefficient (r) and their respective p-values for parasitic EPGs among the techniques and scatterplots were also obtained, to evaluate the linear relationship between the two techniques and demonstrate whether there was a linear trend in the results obtained between them.

All these analyzes were obtained using SPSS (Statistical Package for the Social Sciences) version 25.0, and the graphs were produced in Microsoft Excel version 365.

The difference between the techniques with respect to the EPG was analyzed using Wilcoxon signed-ranks test, i.e., for paired samples that do not follow a normal distribution, at a 5% level of significance for Wilcoxon test using Jamovi® version 2.2 software. Mean and maximum values, standard deviation (SD) and coefficient of variation (CV) were obtained using Microsoft Excel version 365.

## Results

The agreement of diagnoses between the techniques according to the taxa of the parasites was compared using the Kappa coefficient, which revealed substantial agreement in all comparisons performed. In general, with the Mini-FLOTAC technique, a greater number of positive samples for helminths was detected, demonstrating that a performance of Mini-FLOTAC was superior in the detection of parasite forms (Table 1).

**Table 1:** Agreement of the results obtained by parasitological techniques to detect helminth eggs in fecal samples from pigs raised on family farms in Cachoeiras de Macacu, RJ.

**Parasites**	**McMaster**	**Mini-FLOTAC**		**Kappa (CI 95%)**
		**Positive**	**Negative**	
*Ascaris suum*	**Positive**	20	2	0.782 (0.629 - 0.936)
	**Negative**	5	47	
*Trichuris suis*	**Positive**	12	0	0.686 (0.481 - 0.892)
	**Negative**	8	54	
*Strongyloides ransomi*	**Positive**	33	1	0.6547 (0.49 - 0.81)
	**Negative**	12	28	
*Strongyles*	**Positive**	62	1	0.7691 (0.63 - 0.94)
	**Negative**	3	8	

CI: Confidence Interval; k = Kappa. Kappa <0 there wasn´t agreement, 0 to 0.20 poor agreement, 0.21 to 0.40 fair agreement, 0.41 to 0.60 moderate agreement, 0.61 to 0.80 good agreement and 0.81 to 1 almost perfect agreement (Landis & Koch, 1977).

McMaster technique showed higher egg counts of the nematodes *Ascaris suum*, *Trichuris suis* and strongyles, while the Mini-FLOTAC technique showed higher counts of *Strongyloides ransomi*. Parasite loads considered high according to the parameter used here were observed in the mean counts of strongyle eggs obtained by both techniques, as well as in the counts of *A. suum* eggs using McMaster method. Moderate infections were revealed by the mean values of *A. suum* determined by Mini-FLOTAC and *S. ransomi* by both the Mini-FLOTAC and McMaster techniques.

In general, a significant difference between the quantitative techniques was observed in the mean values of EPG of all the helminths (p<0.05). High coefficients of variation in helminth egg counts were observed by both techniques, with the highest coefficients being verified within Mini-FLOTAC technique, except for *A. suum* (Table 2).

**Table 2:** Mean EPG values determined by the two quantitative techniques for the recovery of nematode eggs in feces of pigs raised on family farms.

**Quantitative technique**	***Ascaris suum***			***Trichuris suis***			***Strongyloides ransomi***			**Strongyles**		
	**Mean ±SD**	**CV (%)**	**Max.**	**Mean ±SD**	**CV (%)**	**Max.**	**Mean ±SD**	**CV (%)**	**Max.**	**Mean ±SD**	**CV(%)**	**Max.**
McMaster	988 ± 3090	312.7	18744	37.5 ± 123	328	660	402 ± 1015	252.5	6006	893± 1959	219.4	10989
Mini-FLOTAC	486 ± 1475	303.5	7180	19.5 ± 75.5	387.2	505	404 ± 1512	374.2	12105	696 ± 2508	360.3	20425
Wilcoxon test (p-value)	0.001*			0.035*			0.016*			0.001*		

SD: standard deviation; CV: coefficient of variation; Max.: maximum values of EPG.

* statistically significant (p ≤ 0.05).

Mean of EPG≤100: low parasite load; EPG>100<500: moderate parasite load; EPG≥ 500: high parasite load (Nwafor et al., 2019).

**Figure 1:**
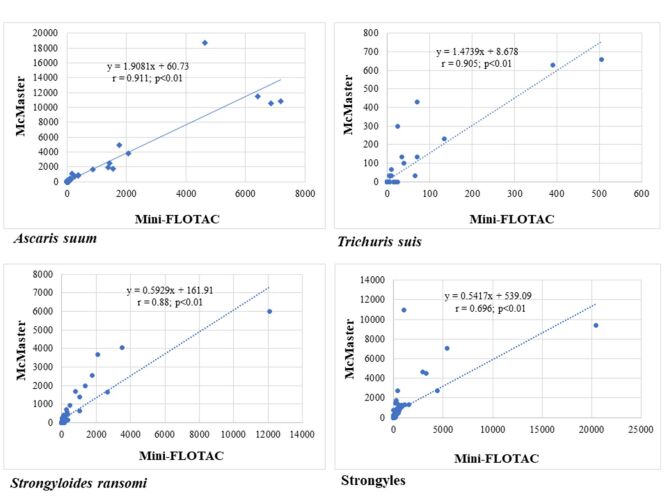
Scatterplots of helminth egg counts, as well as p-value and Pearson's correlation coefficient obtained from analysis of pig fecal samples between Mini-FLOTAC and McMaster techniques.

Higher values of Pearson's linear correlation coefficient (r) were observed between the Mini-FLOTAC and McMaster techniques in relation to the EPG of *A. suum* and *T. suis*, unlike what was evidenced in the EPG of strongyles and *S. ransomi* (Table 1). From the scatterplots it was possible to observe that the points, that is, the EPG values obtained between both techniques tended to approach the straight line mainly in the egg counts of *A. suum* and *T. suis* (Figure 1).

## Discussion

The findings of this analysis indicated that the Mini-FLOTAC technique presented better diagnostic results for helminths in pig fecal samples than the McMaster method. Although the Kappa statistic showed substantial agreement regarding the diagnosis of all parasite taxa, it was still not perfect. A higher number of positive samples were diagnosed using the Mini-FLOTAC technique than with McMaster for all parasite taxa, but mainly for *S. ransomi* and *T. suis*.

It is worth mentioning that this is the first study that compares these techniques applied to pig fecal matter in Brazil, and other studies in the literature that analyzed these techniques with pig feces have not been retrieved. The Mini-FLOTAC has also been shown to be superior to McMaster in the diagnosis of helminths in feces of other host species and in the diagnosis of strongyles in horses in the United States, strongyles and *Strongyloides* in equine and cattle feces in Brazil (Noel et al., 2017; Dias de Castro et al., 2017), *Ascaris lumbricoides* and *Hymenolepis nana* in children’s feces in Argentina (Barda et al., 2014), and strongyles and other nematodes in sheep, goat and rabbit feces (Alowanou et al., 2021). In general, the efficiency of Mini-FLOTAC in the diagnosis of parasites in pig feces was already expected, since its chamber holds a greater volume of fecal suspension (1mL per chamber) than that of the McMaster (0.15 mL per chamber) (Cringoli et al., 2017).

It should be noted that in this study, a Fill-FLOTAC was used with both the Mini-FLOTAC and the McMaster methods. This device facilitates the quantitative parasitological diagnosis in the field, especially in rural areas where suitable infrastructure is rarely available. This is the case of family pig farms usually situated far from large urban centers where most clinical analysis and research laboratories are located. Therefore, we sought to use a method that was potentially applicable in diverse circumstances. Moreover, because the Fill-FLOTAC device is a closed system, it minimizes exposure to potentially zoonotic infectious agents (Lima et al., 2015; Barda et al., 2014; Capasso et al., 2019).

The pigs whose fecal matter was analyzed in this study were raised on small farms that invested little in sanitation management; therefore, moderate to high parasite loads, such as those of strongylids and *A. suum*, were expected. The high EPG counts in the animals’ fecal matter may have concealed marked differences in sensitivity of the quantitative diagnosis of parasites between Mini-FLOTAC and McMaster. However, in the recovery of embryonated eggs of *S. ransomi*, the higher multiplication value of the McMaster method could not surmount the egg counting efficiency of the Mini-FLOTAC technique.

In a comparison of the EPG count obtained by the quantitative techniques with the McMaster was identified a higher mean count for most of the parasite taxa. However, for both techniques, the coefficients of variation showed high values, highlighting different EPG counts in relation to the mean values. This high variation in relation to the average was already expected, since the pigs in the present study came from different family properties that do not follow a standardization in the management of the animals, providing them anthelmintics sporadically and without prior diagnosis. In addition, anthelmintic resistance cannot be ruled out, which may be occurring in some individuals, who end up showing higher EPG values than the others.

On the Mini-FLOTAC these CVs were even larger than on the McMaster, with the exception of *A. suum* EPG. It should be noted that the larger size of the chambers of the Mini-FLOTAC device may have favored a better flotation of nematode eggs, especially those with lower densities and thin-shelled eggs, such as strongyles and *S. ransomi*. In the case of *A. suum*, the CVs presented similar values, that is, the dispersion of the EPG values was practically the same between the techniques. This small difference in the coefficients may have been favored by the high reproductive capacity of the ascarid, that is, the high production and elimination of eggs in the feces, which may have masked differences in sensitivity between the quantitative techniques.

In general, high and low helminth egg counts were verified by both techniques when observing the graphs and correlation coefficients (r), mainly in the parasite taxa that showed the highest values ​​of r. However, it was verified lower r in EPGs of strongyles and *S. ransomi*. Since in the Mini-FLOTAC it was possible to recover and count a greater amount of eggs for these nematodes, as discussed previously.

Despite the satisfactory results obtained with the Mini-FLOTAC in the detection and quantification of helminth eggs in pig feces, the research group that created the technique recommended that its reading chamber be reused about 50 times to confirm its diagnostic effectiveness (Cringoli et al., 2017). In addition to this recommendation, the cost of reading disk was found to be the main disadvantages in performing this technique, since it has to be changed after a period of time.

The differences in EPG values ​​between McMaster and Mini-FLOTAC may be attributed to the lower precision of the McMaster, since this technique uses a reading device that analyzes a smaller volume of fecal suspension. Hence, this increases the correction factor applied to the final EPG count, resulting in lower precision (Alowanou et al., 2021). The McMaster method should therefore be seen as a fecal parasite screening technique to estimate the parasite load. On the other hand, the Mini-FLOTAC, can be used in the future to evaluate the sensitivity of antiparasitic resistance in pigs, especially in those raised on family farms. Such farms rarely follow a regular deworming program, as has been reported previously in parasitic infections of cattle and horses (Dias de Castro et al., 2017).

Nevertheless, the Fill-FLOTAC associated with Mini-FLOTAC proved to be an efficiency methodological tool, since it recovered intestinal parasite forms in pig feces more frequently than the classic McMaster technique. These parasites cause direct losses in terms of animal health and indirect losses to the farmer. However, in view of the pioneering nature of this study in the diagnosis of parasitic agents in pig feces, further research with this technique is recommended. This includes different flotation solutions and larger sample sizes from pigs subjected to diverse sanitation practices to assess the sensitivity, specificity and predictive values in animals with distinct infection profiles.
